# Reliability and measurement error of frontal and horizontal 3D spinal motion parameters in 219 patients with chronic low back pain

**DOI:** 10.1186/s12998-016-0092-0

**Published:** 2016-04-04

**Authors:** Steen Harsted, Rune M. Mieritz, Gert Bronfort, Jan Hartvigsen

**Affiliations:** Institute of Sports Science and Clinical Biomechanics, University of Southern Denmark, Campusvej 55, Odense M, DK-5230 Denmark; Integrative Health & Wellbeing Research Program, Center for Spirituality & Healing, Academic Health Center, University of Minnesota, 420 Delaware Street SE, MMC-505, 612-301-9005 Room B296-1, Minneapolis, MN 55455 USA; Nordic Institute of Chiropractic and Clinical Biomechanics, University of Southern Denmark, Campusvej 55, Odense M, DK-5230 Denmark

**Keywords:** Low Back Pain, Subgroup analysis, Motion analysis, Biomechanics, Reproducibility, Reliability

## Abstract

**Background:**

In order for measurements to be clinically useful, data on psychometric conditions such as reliability should be available in the population for which the measurements are intended to be used. This study comprises a test-retest design separated by 7 to 14 days, and evaluates the intra and interrater reliability of regional frontal and horizontal spinal motion in 219 chronic LBP patients using the CA6000 Spine Motion Analyzer. In addition, it compares these results on the frontal and horizontal plane with previously published results on the sagittal plane. 219 individuals with chronic mechanical LBP, classified as either Quebec Task Force group 1, 2, 3 or 4 were included, and kinematics of the lumbar spine were sampled during standardized spinal lateral flexion and rotation motion using a 6-df instrumented spatial linkage system. Test-retest reliability and measurement error were evaluated using intraclass correlation coefficients ICC_(1,1)_ and Bland-Altman limits of agreement (LOAs).

**Results:**

The reliability analysis based on the whole study sample showed ICC_(1,1)_ coefficients varying between 0.68 and 0.73 for the frontal plane and 0.33 and 0.49 for the horizontal plane. Relatively wide LOAs were observed for all parameters. Reliability measures in patient subgroups ICC_(1,1)_ ranged between 0.55 and 0.81 for the frontal plane and 0.28 and 0.69 for the horizontal plane. Greater ICC_(1,1)_ coefficients and smaller LOA were observed when patients were examined by the same examiner, had a stable pain level between tests, and were male. ROM measurements were more reliable in patients with a BMI higher than 30, and measurements on patients with LBP and leg pain showed higher reliability and smaller measurement error in all parameters except for the jerk index.

**Conclusion:**

Frontal plane measurements obtained using the CA6000 Spine Motion Analyzer are sufficiently reliable to be used for group comparisons but not individual comparisons. Measurements in the horizontal plane can be used for neither group nor individual comparisons.

## Background

It is widely assumed that Low Back Pain (LBP) with mechanical pain behavior is significantly influenced by biomechanical factors [1–4]. Therefore kinematics obtained using noninvasive 3-dimensional (3D) regional lumbar spinal motion instruments may be of value in generating functional diagnoses, evaluating the mechanisms of therapies, and prescribing specific rehabilitation programs [1, 2, 5], as for example in a recent clinical study on mobilization with movements in mechanical LBP patients [6].

In order for measurements to be clinically useful, data on psychometric conditions such as reliability should be available in the population for which the measurements are intended to be used. [7, 8]. Most reliability studies evaluating 3D regional lumbar instruments have been performed using convenience samples of asymptomatic individuals [9], and therefore little is known about the reliability of these methods when they are used on clinically relevant populations. In addition, it is unclear if or how movement characteristics may differ among subgroups of LBP patients, which in turn may affect reliability.

We recently presented values and estimates for reliability and measurement error of sagittal plane spinal motion using a 3D lumbar spinal motion instrument called the CA6000 Spine Motion Analyzer in chronic and care seeking LBP patients and further stratified the sample into subgroups based on body mass index (BMI), gender, differences in pain level, and Quebec Task Force classification [10]. We found that the estimates differed substantially between the subgroups with intraclass correlation coefficients (ICC_(1,1)_) ranging from 0.34 to 0.77 [10], and a number of characteristic subgroup patterns were observed. These patterns where in general greater ICC_(1,1)_ coefficients and smaller limits of agreement (LOA) when patients were examined by the same examiner, had a BMI below 30, had a stable pain level between test one and two, were men and could be classified in the Quebec Task Force classifications Group 1.

Previous studies using the same type of 3D regional lumbar instruments have found measures of flexion/extension and lateral bending movements to be more reliable than axial rotation [11–16] possibly related to technical difficulties with measurements performed in the horizontal plane, e.g. slipping of the mounting plates across the skin [12].

The overall aim of the present study is to evaluate the reliability of regional frontal and horizontal spinal motion in 219 chronic LBP patients using the CA6000 Spine Motion Analyzer. In addition, we aim to discuss potential differences between frontal and horizontal plane motion, and compare results on the frontal and horizontal plane with previously published results on the sagittal plane [10].

We hypothesize that frontal plane movements show better reliability and have smaller measurement error when compared to similar measures in the horizontal plane. In addition, that reliability and measurement errors in the subgroups (same or different examiner, BMI below or above 30, stable or unstable pain, gender, low back pain with or without leg pain) will follow the same pattern as in the sagittal plane motion [10].

## Methods

### Study population

During a period of 3 years from 2001 to 2004, 219 subjects were recruited for inclusion in a randomized clinical trial at the Wolfe Harris Center for Clinical Studies at Northwestern Health Science University, Minneapolis, MN, USA [17]. Recruitment was done through local newspaper advertisements, community posters, postcard mailings, and followed by an initial screening conducted by telephone. Eligible participants included individuals from 18 to 65 years of age who had a primary complaint of mechanical low back pain of at least 6-week duration and could be classified as Quebec Task Force 1,2,3 or 4 [18].

Mechanical LBP was defined as pain with no specific identifiable etiology but reproducible by back movements or provocations tests. Exclusion criteria were previous lumbar spine fusion surgery, progressive neurological deficits, aortic or peripheral vascular disease, pain scores of less than 3 points (on a 0–10 point scale), ongoing treatment for back pain by other healthcare providers, or participation in pending or current litigation.

The study was approved by the institutional review boards of the Northwestern Health Sciences University, the Minneapolis Medical Research Foundation, and the University of Minnesota. Written informed consent was obtained from all the study participants.

### Test procedures

The test-retest design was conducted as part of the baseline examination for a randomized clinical trial [17] and included two visits separated by 7 to 14 days. At the first visit, the participants completed a self-administered questionnaire on health history and demographics, data regarding the participants’ height and weight were recorded, and a complete neurologic examination, orthopedic tests and manual static and motion palpation of the lumbar spine and lower extremities were performed. Licensed chiropractic and medical clinicians conducted the physical examination. Nine blinded, trained and certified research clinicians conducted the objective 3D movement measurements described in detail in this article.

Subjects who qualified for the clinical trial and agreed to participate were scheduled for a second baseline visit in the clinic 7 to 14 days later, at which time the 3D motion measurements were repeated.

### Measurement protocol

#### Instrumentation

Kinematics of the lumbar spine movements in the frontal and horizontal plane were sampled following a standardized test protocol using a 6-df instrumented spatial linkage system with a sampling rate of 100 Hz (CA6000 Spine Motion Analyzer; OSI, Union City, CA, USA).

### Attachment and procedure

Each participant wore a loose T-shirt and pants. We have previously described the attachment of the measuring device [10]. After the participant had performed a backward and forward bending they were given the following verbal instruction to perform a left and right turning “Again, for this one, find your neutral position with your arms over your chest. Turn your torso to your left and then to your right and the back to neutral. Be sure to turn from your waist and to go as far as you can go in both directions at your own pace without pausing”. The instructor simultaneously showed the movement as he was giving the instructions. The patient was allowed to practice the movement once, and the equipment was checked before recording started. The procedure was repeated until two valid ROMs were recorded (The total ROM of one trial should be within 4° of a second trial). The outcomes of the two trials were averaged and used for the analysis.

When the left and right turning had been performed the participant was asked to stand straight and was then given the following instructions both verbally and visually “Ok, this time find your neutral position with your palms on the side of your thighs. Slide your hand down your leg as you lean to the left, then to the right, and then back to neutral. Be sure to go as far as you can go in both directions at your own pace without pausing”. The patient was allowed to practice the movement once, and the equipment was checked before recording started.

The procedure was repeated until two valid measurements had been recorded (The total ROM between two should be within 4° of each other). The outcomes of the two trials were averaged and used for the analysis.

### Data processing and analysis

A comprehensive description of data processing procedure including MATLAB programming, computerized trial selection algorithm has been published elsewhere [10]. This MATLAB program was also used to reduce the 3D data into numbered motion parameters, that where selected on the current study hypothesis, previous study findings [5, 19, 20] and clinical experience.

The following motion parameters were selected for the frontal plane:

1. ROM lateral flexion defined as the total angular range of spine motion in the frontal plane expressed in degrees from maximum left to right.
2. Mean velocity lateral flexion (°/s) defined as the central limit theorem as the average angular speed from maximum left to right.
3. Phase-plot area lateral flexion (°^2^/s) defined as the area composed by the phase-plot of lateral flexion left to right angular motion versus velocity. Phase-plot area was calculated based on cross-product calculations between vectors drawn from neutral position to each coordinate point.
4. Jerk index lateral flexion defined as maximum lateral flexion left to right as the mean spectral frequency of the first derivative of the angular acceleration signal multiplied by movement duration. In this way this parameter calculates the number of changes in acceleration and thereby indicates the smoothness of the motion [10].

The following motion parameters were determined for the horizontal plane:

1. ROM rotation defined as the total angular range of spine motion in the horizontal plane expressed in degrees from maximum left to right.
2. Mean velocity rotation (°/s) defined as the central limit theorem as the average angular speed from maximum left to right
3. Phase-plot area rotation (°^2^/s) defined as the area composed by the phase-plot of rotation left to right angular motion versus velocity. Phase-plot area was calculated based on cross-product calculations between vectors drawn from neutral position to each coordinate point.
4. Jerk index rotation defined as maximum rotation left to right as the mean spectral frequency of the first derivative of the angular acceleration signal multiplied by movement duration.

### Statistical analysis

The statistical analysis was performed using the statistical package Stata software (StataCorp, College Station, TX, USA) version 13 on a personal computer. Taxonomy, terminology, and definitions related to reliability and measurement error are based on Consensus-based Standards for the Selection of Health Measurement Instruments (COSMIN) [21].

Based on the study design each target was rated by a different set of the nine examiners (considered to be randomly selected from a larger population of examiners) and because we aim to generalize to individual ratings the Intraclass Correlation Coefficients (ICC_1,1_) were calculated to assess reliability [22]. The intraclass correlation coefficient (ICC_(1,1)_) [22] and Bland-Altman limits of agreement (LOAs) [23] with a 95 % confidence interval (CI) were calculated to assess test-retest reliability and to evaluate measurement error. Paired t-tests were used to detect systematic bias between tests sessions using the trial means.

We used Bland-Altman plots [23] and correlation analysis between absolute differences and mean values to test the assumption of normally distributed and homoscedastic data. No heteroscedastic relationships were found.

We stratified into subgroups based on pain distribution, (back and leg pain versus back pain only) by collapsing Quebec diagnostic groups 2, 3, and 4 versus Quebec diagnostic group 1 [18]. The BMI cutoff point was based on the World Health Organization for the classification of overweight (i.e., BMI ≥ 30) [24] and the unstable pain subgroup was defined as patients with a change in score (the numerical rating scale) between test and retest of ±2 points or more [25].

## Results

Six hundred thirty individuals were considered for inclusion in the study, of these 329 were not included because they did not meet the inclusion criteria specified in the primary study [17]. Of the 301 included, 22 did not complete both assessments and 59 were excluded due to technical problems during the testing (defects in the measuring device (*N* = 6) and a database storing error (*N* = 53)). Reasons for dropout were recorded as follows: refused to participate (*N* = 9), personal conflict (*N* = 3), increase in musculoskeletal pain (*N* = 3), insufficient time (*N* = 2), unknown reasons (*N* = 2), competing co-morbidity (*N* = 1) and change of address (*N* = 1)), leaving 220 complete datasets of which one was excluded because the participant had performed the side-bending movements in reverse order and thus a total of 219 datasets were available for analysis. The clinical characteristics of the study population are showed in (Table 1).

**Table 1:** Subject characteristics for the present group of male and female LBP patients

		Male (*n* = 87)		Females (*n* = 132)		
	Mean (SD)	95 % CI	Range	Mean (SD)	95 % CI	Range
Age (years)	44.7 (11.3)	[42.7 - 47.1]	22-64	45.9 (11)	[43.9 - 47.8]	22-65
Height (cm)	178 (6.3)	[176–179.3]	164-196	163.3 (8.3)	[161.3 - 164.7]	133-183
Weight (kg)	89.2 (15.2)	[87.2 - 92.4]	66-159	75.1 (16.7)	[73.1 - 77.9]	45-133
BMI	28.1 (4.7)	[26.1 – 34]	19.3-49.6	28.2(6.2)	[26.2 - 29.3]	18.7-47.2
Activity level	2.4 (1.3)	[0.4 - 2.9]	0-4	2.4 (1.2)	[0.4 - 2.6]	0-4
Past episodes of back pain	3.4 (0.6)	[1.4 - 4.1]	1-4	3.2(0.9)	[1.2 - 3.5]	0-4
S1 LBP past week	5.1 (1.6)	[3.1 - 6.2]	3-9	5.2 (1.6)	[3.2 - 5.5]	3-10
S2 LBP past week	5.3 (1.6)	[3.3 - 6.4]	2-8	5.1 (1.8)	[3.1 - 5.4]	2-10
S1 LP past week	2.1 (2.4)	[0.1 - 2.5]	0-9	2.3 (2.6)	[0.3 - 2.7]	0-10
S2 LP past week	2.0 (2.5)	[0–2.4]	0-8	2.1 (2.5)	[0.1 - 2.5]	0-10
S1 Roland Morris (0–23)	8.1 (4.4)	[6.1 - 9.8]	2-17	8.8 (4.3)	[6.8 - 9.5]	1-21
S2 Roland Morris (0–23)	8.0 (5.0)	[6–9.7]	0-23	8.7 (4.9)	[6.7 - 9.5]	0-21

CI, confidence interval; SD, standard deviation

Activity level: *Engaged in exercise or sports activities in the past month? (0 = I do not engage in exercise or sports, 1 = Less than once a week, 2 = Once a week, 3 = 2 or 3 times per week, 4 = 4 times or more per week)*

Past episodes of LBP: *An episode is a week with at least some LBP (0 = None, 1 = 1–2 episodes, 2 = 3–5 episodes 3 = More than 5 episodes, 4 = 1 single episode of continuous LBP)*

(S) *= session,* (LP) *= leg pain,* (LBP) *= low back pain (measured on a numerical pain scale “one to ten”, ten being the worst possible pain)*

There were no differences in baseline characteristics between the final sample of 219 individuals and the 82 individuals not available for analysis with respect to BMI, gender, duration of pain, or depression score (data not shown). The individuals not available for analysis were however slightly younger (median age of 43 versus 47 years).

A statistically significant difference between session 1 and 2 was found in 3 out of 4 of the motion parameters for rotation: ROM (7.6 % higher at session 2, *p* = 0.009), Rotation mean velocity (13.6 % higher at session 2, *p* < 0.0001), Phase-plot area (15.3 % higher at session 2, *p* = 0.0002). For lateral flexion, a statistical difference between the two sessions was found in 2 out of the 4 motion parameters: Lateral flexion mean velocity (4.6 % higher at session 2, *p* = 0.043) and phase plot area (4.8 % higher at session 2, *p* = 0.045) (Table 2).

**Table 2:** Motion parameters recorded during voluntary lumbar frontal and horizontal plane motion

	Parameter	Session 1			Session 2			
		Mean (SD)	Min	Max	Mean (SD)	Min	Max	*p value*
Frontal plane:	ROM lateral flexion (°)	48.4 (9.7)	19.9	73.4	48.5 (7.8)	20.5	81.2	0.838
	Lateral flexion mean velocity (°/sec)	15.2 (6)	4.9	36.9	15.9 (6.1)	4.5	36.1	0.043
	Phase-plot Area lateral flexion(°/(°/sec))	3179 (1477)	509	7964	3333 (1458)	493	7532	0.045
	Jerk index lateral flexion	11.3 (5.2)	3.6	36.3	10.8 (5.3)	3	34.9	0.052
Horizontal plane:	ROM rotation (°)	35.6 (11.6)	5.9	62.6	38.3 (10.6)	5.1	63.8	0.0009
	Rotation mean velocity (°/sec)	11.7 (5.3)	1.6	28.7	13.3 (5.8)	1.2	35.2	<0.0001
	Phase-plot Area rotation(°/(°/sec))	1826 (1083)	57	5254	2106 (1182)	40	6042	0.0002
	Jerk index rotation	11.99 (4.53)	4.9	29.8	11.56 (4.77)	3.8	43.2	0.1358

Min, minimum; Max, maximum; ROM, range of motion; SD, Standard deviation

Means, standard deviations, range and *P*-value (*t* test) for all 219 patients flexion motion measurements with the OSI Spine Motion Analyzer

All session values are the average of two subsequent trials with a ROM difference of 4° or less

The reliability analysis based on the whole study sample showed ICC_(1,1)_ coefficients varying between 0.68 and 0.73 for the frontal plane and 0.33 and 0.49 for the horizontal plane. Furthermore relatively wide LOA values (e.g. ROM ranged from −15.4 to 15.2° in lateral flexion and −25.3 to 20.1° in rotation) were found for all motion parameters in both frontal and horizontal plane movements (Table 3).

**Table 3:** Overall reliability lumbar spinal motion parameters in the frontal and horizontal planes

	Parameter (*n* = 219)	Reliability			Agreement	
		ICC(1,1)	(95 % CI)		95 % LOA	SEM
Frontal plane:	ROM (°) lateral flexion	0.68	0.61	0.75	−15.4 - 15.2	8.02
	Lateral flexion mean velocity (°/sec)	0.71	0.64	0.77	−9.7 - 8.4	5.11
	Phase-plot Area lateral flexion (°/(°/sec))	0.7	0.63	0.77	−2368 - 2060	1228.91
	Jerk index lateral flexion	0.73	0.66	0.79	−7.06 - 8.08	4.48
4Horizontal plane:	ROM (°) rotation	0.44	0.33	0.54	−25.3 - 20.1	3.54
	Rotation mean velocity (°/sec)	0.53	0.43	0.62	−11.8 - 8.7	4.08
	Phase-plot Area rotation (°/(°/sec))	0.5	0.4	0.6	−2455 - 1896	806.82
	Jerk index rotation	0.58	0.49	0.67	−7.93 - 8.79	3.54

CI, confidence interval; ICC, intraclass correlation coefficients; LOA, Bland-Altman limits of agreement; ROM, range of motion; SEM, standard error of measurement

Reliability data from the subgroup analyses ICC_(1,1)_ ranged from 0.28 to 0.69 for horizontal plane and 0.55 to 0.81 for frontal plane movements. For all parameters frontal plane movements showed higher reliability scores than movements in the horizontal plane (Table 4).

**Table 4:** Spinal motion reliability and measurement error in the frontal and horizontal planes for LBP patients divided into subgroups

	Motion Parameter	Statistical parameter	Subgroups									
			Same ex.	Different ex.	BMI <30	BMI > 30	Pain (s)	Pain (u)	Male	Female	Grp. 1^a^	Grp. 2,3,4^a^
		Number of subjects	89	130	146	73	161	58	87	132	148	71
Frontal plane:	ROM lateral flexion	ICC(1,1)	0.75	0.63	**0.55**	**0.81**	0.68	0.67	0.65	0.69	0.64	0.74
		95 % CI	[0.66-0.84]	[0.53-0.73]	[0.44-0.67]	[0.74-0.89]	[0.60-0.77]	[0.53-0.81]	[0.52-0.77]	[0.6-0.78]	[0.55-0.74]	[0.64-0.85]
		LOA_LL(degree)	−14	−16.2	−17	**−11.9**	−14.1	**−18.8**	−15	−15.8	−16.4	−13.3
		LOA_UL(degree)	12.2	**17.1**	16.6	**12**	14.5	16.9	14.4	15.8	15.9	13.7
	Mean velocity lateral flexion	ICC(1,1)	0.7	0.71	0.69	0.73	0.75	**0.58**	0.74	0.68	0.68	**0.76**
		95 % CI	[0.59-0.8]	[0.63-0.8]	[0.61-0.78]	[0.62-0.84]	[0.68-0.81]	[0.4-0.75]	[0.65-0.84]	[0.58-0.77]	[0.59-0.76]	[0.66-0.86]
		LOA_LL(ratio)	−10.1	−9.3	−10.1	**−8.9**	−9.4	**−10.3**	−9	−10.1	−9.7	−9.6
		LOA_UL(ratio)	7.6	8.9	9.1	**7**	7.8	**10.1**	7.8	8.8	8.7	7.8
	Phase-plot Area lateral flexion	ICC(1,1)	0.74	**0.67**	**0.67**	**0.75**	0.72	0.65	0.72	0.68	0.68	0.73
		95 % CI	[0.65-0.84]	[0.58-0.77]	[0.58-0.76]	[0.65-0.85]	[0.65-0.8]	[0.5-0.8]	[0.62-0.82]	[0.59-0.77]	[0.6-0.77]	[0.63-0.84]
		LOA_LL(ratio)	−2265	−2381	−2472	−2127	−2253	**−2679**	**−1982**	−2598	−2358	−2398
		LOA_UL(ratio)	**1538**	**2460**	2253	1641	1943	2375	1706	2269	2112	1961
	Jerk index lateral flexion	ICC(1,1)	0.73	0.73	0.75	0.69	0.74	0.69	**0.80**	**0.60**	0.76	0.61
		95 % CI	[0.63-0.82]	[0.64-0.81]	[0.67-0.81]	[0.57-0.81]	[0.67-0.81]	[0.56-0.83]	[0.73-0.88]	[0.5-0.71]	[0.69-0.83]	[0.47-0.76]
		LOA_LL(ratio)	−6.5	−7.35	−7.28	−6.56	**−6.19**	**−9.08**	−7.27	−6.95	−7.39	−6.24
		LOA_UL(ratio)	**8.83**	**7.47**	7.78	8.64	7.96	8.03	8.39	7.91	7.71	8.72
Horizontal plane:	ROM rotation	ICC(1,1)	**0.69**	**0.28**	0.36	0.56	0.44	0.41	0.45	0.43	0.35	0.63
		95 % CI	[0.57-0.8]	[0.12-0.44]	[0.21-0.5]	[0.4-0.72]	[0.32-0.57]	[0.19-0.62]	[0.28-0.61]	[0.3-0.57]	[0.21-0.49]	[0.49-0.77]
		LOA_LL(degree)	**−18.8**	**−29**	−26.6	−22.8	−24.9	−26.3	−26.5	−24.6	−27.8	−19.3
		LOA_UL(degree)	**14.2**	**23.3**	20.8	18.6	20.9	17.6	20.7	19.7	21.7	15.9
	Mean velocity rotation	ICC(1,1)	0.56	0.51	0.51	0.57	0.53	0.52	0.54	0.52	**0.5**	**0.59**
		95 % CI	[0.42-0.71]	[0.38-0.63]	[0.39-0.63]	[0.41-0.72]	[0.42-0.64]	[0.33-0.71]	[0.4-0.69]	[0.39-0.64]	[0.37-0.62]	[0.44-0.74]
		LOA_LL(ratio)	−10.8	−12.4	**−12.6**	**−10**	−11.7	−12.3	−11.8	−11.9	−12	−11.3
		LOA_UL(ratio)	**7.4**	**9.6**	9.3	7.5	8.8	8.8	9.1	8.6	8.6	9.2
	Phase-plot Area rotation	ICC(1,1)	**0.68**	**0.4**	0.45	0.6	0.47	0.57	0.50	0.5	0.45	0.6
		95 % CI	[0.57-0.79]	[0.26-0.55]	[0.32-0.58]	[0.45-0.75]	[0.35-0.59]	[0.39-0.74]	[0.34-0.66]	[0.37-0.63]	[0.32-0.58]	[0.44-0.75]
		LOA_LL(ratio)	**−1847**	**−2789**	−2673	−1970	−2460	−2434	−2289	−2566	−2524	−2311
		LOA_UL(ratio)	**1254**	**2253**	2014	1612	1989	1629	1760	1987	1863	1963
	Jerk index rotation	ICC(1,1)	0.58	0.58	0.65	**0.37**	0.59	0.53	**0.66**	0.44	0.6	0.49
		95 % CI	[0.44-0.72]	[0.46-0.69]	[0.55-0.74]	[0.17-0.57]	[0.49-0.69]	[0.35-0.72]	[0.54-0.78]	[0.31-0.58]	[0.5-0.7]	[0.31-0.67]
		LOA_LL(ratio)	−7.8	−7.96	−7.99	−7.73	−7.92	−7.97	**−9.09**	**−7.1**	−8.33	**−7.1**
		LOA_UL(ratio)	9.7	**8.14**	8.28	**9.75**	9.01	8.24	8.95	8.63	8.98	8.41

BMI, body mass index; CI, confidence interval; ex., examiner(s); grp., group; ICC, intraclass correlation; LBP, low back pain; LL, lower limit; LOA, Bland-Altman limits of agreement; Pain (s), numerical rating scale maximum change ±1; Pain (u), numerical rating scale change ± 2 or more; ROM, range of motion; UL, upper limit

Note: Maximum and minimum values in each row are in bold type

^a^ Quebec Task Force classifications 1 versus 2, 3 and 4

Participants with higher BMI (BMI > 30) had higher ICC_(1,1)_ values and lower LOA scores between the two sessions in all parameters except the jerk index. Participants examined by the same examiner showed higher ICC_(1,1)_ coefficients and smaller LOA values between the two sessions in all motion parameters except the Jerk index which showed identical ICC_(1,1)_ coefficients in the inter and intra examiner analysis.

Participants with unstable pain showed a trend towards lower ICC_(1,1)_ coefficients and higher LOA scores between the sessions for the frontal plane movements, whereas the horizontal plane movements showed mixed results – with ICC_(1,1)_ coefficients being slightly lower for ROM, mean velocity and jerk index and higher for Phase-plot area.

Finally, participants with LBP and leg pain had less variation in ROM, mean velocity and Phase-plot area between the sessions, but a lower ICC _(1,1)_ coefficient for the Jerk index when compared to participants with LBP only.

## Discussion

We found systematic differences between the test and retest session for three of the four motion parameters in the horizontal plane and in two of the four motion parameters in the frontal plane with one further parameter showing borderline significant differences between the two sessions. These differences most likely indicate the presence of a learning effect caused by patients’ habituation to the instrument and the testing situation.

Our hypotheses were confirmed as we found that frontal plane movements yield higher ICC_(1,1)_ coefficients on all measured parameters and across all subgroups when compared to the horizontal plane. Frontal plane motions, exhibit moderate to good reliability with ICC_(1,1)_ values, ranging between 0.55 and 0.8 [26]. Therefore, frontal plane movement data obtained using the CA6000 Spine Motion Analyzer can be used for group comparisons such as those used in research but not for individual comparisons in the clinical setting. All calculated ICC_(1,1)_ values for horizontal motion parameters range from poor to moderate[26], and no single parameter in the horizontal plane was found with an ICC_(1,1)_ value above 0.69. Finally, LOA where found to be relatively wide. Although frontal plane motion parameters have slightly smaller LOA, we deem the LOA of both planes to be too wide for the CA6000 Spine Motion Analyzer to be used in clinical practice. Therefore, we cannot recommend the use of horizontal movement parameters obtained with this device for neither group nor individual comparisons, and subgroup patterns for horizontal motion should be interpreted with extra care [26, 27]. We believe, like Dvorak et al.[12], that the lower ICC_(1,1)_ values obtained in the horizontal plane using the CA6000 Spine Motion Analyzer may be caused by slipping of the mounting plates across the skin during rotation of the trunk. It should be noted that other types of kinematic devices have been recommended for all planes of trunk movement except extension by other authors [28, 29].

We further hypothesized that in general greater ICC_(1,1)_ coefficients and smaller LOA would be found when patients were examined by the same examiner, had a BMI below 30, had a stable pain level, were men and could be classified in the Quebec Task Force classifications Group 1. We found that some of the patterns were as we had hypothesized in that patients, who were examined by the same examiners, had a stable pain level, and were men showed less variation between the two test sessions and had in general smaller LOA. This pattern is consistent across all three planes of motion, and it is therefore a general finding that intraexaminer measurements and measurements made on men and pain stable patients are more reliable than interexaminer measurements, and measurements made on women and patients who have unstable pain.

Among patients with a BMI above 30 and patients with both LBP and leg pain, we unexpectedly found less variation, and smaller LOA between the sessions compared to findings from patients with a BMI below 30, and among patients who had LBP without leg pain. We therefore rejected our second hypothesis as a whole because only some of the subgroup comparisons showed the expected patterns.

We observed greater ICC_(1,1)_ coefficients, and smaller LOA in ROM for all three planes of motion among patients with a BMI above 30. We speculate that this may be because the larger body mass generally limits motion and thus these measurements likely reflect body size rather than spine function. A secondary comparative analysis on mean ROM in the frontal plane comparing patients with a BMI above 30 to patients with a BMI below 30 did indeed show that patients with higher BMI had lower ROM 44.5(9.5) (mean (SD)) versus 50.4(8.0), *p* < 0.0001 (paired *t*-test)). Another secondary analysis showed that patients who reported different pain scores between session 1 and 2, also had more changes in ROM between the two sessions if their BMI was below 30 (ICC_(1,1)_ value 0.43), whereas patients with unstable pain and a high BMI had more similar ROM values between the two sessions (ICC_(1,1)_ value 0.84).

On average people with LBP have reduced lumbar ROM [30]. Our secondary analysis suggests that patients with a BMI above 30 represents a subgroup of LBP patients with even further reduced ROM. We suggest that a ROM limiting factor specific to this subgroup could be excess intraabdominal fat tissue reducing the ROM through compression. This would explain why ROM measurements on patients with BMIs above 30 appear to be quite stable even though their pain level has changed in between sessions. Another possible explanation, could be that the findings are caused by a flaw in the measurement device related to measuring ROM on patients with BMI´s above 30, i.e. increased slipping of the mounting plates caused by extra subcutaneous fat, causing the device to yield wrong but consistent results. More research investigating lumbar ROM among subgroups of LBP patients using other types of devices than the CA6000 Spine Motion Analyzer is needed to clarify this further.

Unexpectedly, we found less intersession variation among patients with both low back and leg pain. For these patients ICC_(1,1)_ coefficients were between 0.05 to 0.28 higher than those found for patients with LBP only. A possible explanation for this phenomenon could be that patients with leg pain represent a more homogeneous population with regard to what is causing their pain.

A further comparison with our previous findings [10] revealed nearly identical ICC_(1,1)_ values for the sagittal and frontal plane ROM, mean velocity and the phase-plot, when looking at patients with LBP only. However, the same comparison made among patients with LBP and leg pain revealed ICC_(1,1)_ values to differ substantially more between the two planes of motion, but always with the frontal plane having the highest ICC_(1,1)_ values. Together these findings indicate that when measuring patients using the CA6000 Spine Motion Analyzer, results will be equally reliable for the frontal and sagittal plane if the patients suffer from back pain without leg pain, but if the patients suffer from LBP and leg pain, the results will be more reliable for the frontal plane.

The jerk index is a particularly noteworthy parameter because it captures the quality and smoothness of the movements and not only the range. Among several observations, we have chosen three to be of particular interest. Firstly, with identical or very similar intra and interexaminer ICC_(1,1)_ coefficients the jerk index appears to be almost unaffected by interexaminer bias. This is a consistent finding across all three motion planes [10]. Secondly, while Jerk index ICC_(1,1)_ values in the horizontal plane ranged from poor to low-end moderate, values in the frontal plane ranged from high-end moderate to good. Furthermore all of the ICC_(1,1)_ values in the frontal plane were also notably higher than the previously published results from the sagittal plane [10] – with the lowest difference being in the males (0.80 in the frontal plane and 0.70 in the sagittal plane), and the highest difference being in the BMI > 30 group (frontal plane 0.69 and sagittal plane 0.41). This indicates that when using the Jerk Index parameter as a base-line measurement, an outcome indicator or both, frontal plane measurements will be more reliable. Thirdly, the jerk index is the only motion parameter that showed the same subgroup pattern across all three planes of motion. We therefore speculate that while ROM, Phase plot and Mean velocity may be closely related to biomechanical factors, which change in subgroups depending on the plane examined, the jerk index may instead reflect a component of locomotion that is less dependent on biomechanical factors. Such a component could be biological, i.e. a neuromuscular control mechanism or it could reflect a psychological mechanism such as fear avoidance. Adding a dynamical systems approach to study movement control and coordination as proposed by B. Spinelli et al. [31] may contribute to a deeper understanding of this component of locomotion.

Previous research has shown the jerk index to change significantly and differently depending on whether patients receive spinal manipulation or exercise [32]. Possibly the jerk index reflects an underlying principle which is affected differently by these two commonly used treatment interventions. Previous Jerk index measurements in clinical trials have however been made in the sagittal plane [32], and results from our current analysis show Jerk index measurements to be notably more reliable when made in the frontal plane.

### Study limitations

The CA6000 Spine Motion Analyzer has previously been described in detail and verified for precision and accuracy [15, 33–37]. A majority of these studies have found the device to have high to excellent accuracy and precision in both horizontal and frontal plane motion. A single exception to these findings is however made by Christensen, who found the device to have very high precision but less than acceptable accuracy. Christensen found the accuracy for horizontal and frontal plane signals such as those obtained in the current study to be ranging from 6.0 to 11.5 % for frontal plane signals and from 7.0 to 10.3 % for horizontal plane signals [33].

To reduce complexity and data abundance, the recorded 3D spinal motion data were reduced into a number of separate motion parameters. The selected parameters could be considered as reductionist models to achieve descriptive measures of complex spinal movement patterns in patients with LBP at the functional level. Obviously, selecting some parameters and certain ways to analyze means that other types of important kinematic information may remain uncovered in the data set.

This study has focused on single-plane analysis of the lumbar spine; a combined plane movement analysis may yield different results, which could be of clinical relevance [38].

Use of over the counter pain medication and changes in physical activity may have changed between the two sessions, but were not considered.

This study reports inter-session reliability, with the two sessions 7–14 days apart. Intra session reliability was not considered.

In addition, these results cannot be extrapolated to other technologies.

## Conclusions

Using the CA6000 Spine Motion Analyzer, frontal plane measurements are sufficiently reliable to be used for group comparisons but not individual comparisons. Measurements in the horizontal plane can be used neither for group nor individual comparisons.

Greater ICC_(1,1)_ coefficients and smaller LOA were observed when patients were examined by the same examiner, had a stable pain level between tests, and were male.

We found that ROM measurements were more reliable in patients with a BMI higher than 30, and measurements on patients with LBP and leg pain showed higher reliability and smaller measurement error in all parameters except for the jerk index.

The jerk index appears to be almost unaffected by interexaminer bias regardless of the examined motion plane, and it is more reliable when it is measured in the frontal plane. The jerk index is the only motion parameter to have a consistent subgroup pattern across all three planes of motion.
